# Hexabromocyclododecane (HBCD) Stereoisomers in U.S. Food from Dallas, Texas

**DOI:** 10.1289/ehp.1204993

**Published:** 2012-05-31

**Authors:** Arnold Schecter, David T. Szabo, James Miller, Tyra L. Gent, Noor Malik-Bass, Malte Petersen, Olaf Paepke, Justin A. Colacino, Linda S. Hynan, T. Robert Harris, Sunitha Malla, Linda S. Birnbaum

**Affiliations:** 1 University of Texas School of Public Health, Dallas, Texas, USA; 2 National Center for Environmental Assessment, U.S. Environmental Protection Agency, Arlington, Virginia, USA; 3 Eurofins Gfa GmbH Laboratory, Hamburg, Germany; 4 Department of Environmental Health Sciences, University of Michigan School of Public Health, Ann Arbor, Michigan, USA; 5 Department of Clinical Sciences and Department of Psychiatry, University of Texas Southwestern Medical Center, Dallas, Texas, USA; 6 National Cancer Institute, National Institutes of Health, Department of Health and Human Services, Washington, DC, USA; 7 National Institute of Environmental Health Sciences, National Institutes of Health, Department of Health and Human Services, Research Triangle Park, North Carolina, USA

**Keywords:** Dallas, Texas, food, HBCD, hexabromocyclododecane, stereoisomers

## Abstract

Background: Hexabromocyclododecane (HBCD) is a brominated flame retardant used in polystyrene foams in thermal insulation and electrical equipment. The HBCD commercial mixture consists mainly of α, β, and γ stereoisomers. Health concerns of HBCD exposure include alterations in immune and reproductive systems, neurotoxic effects, and endocrine disruption. Stereoisomer-specific levels of HBCD have not been measured previously in U.S. food.

Objectives: We measured HBCD stereoisomer levels in U.S. foods from Dallas, Texas, supermarkets.

Methods: Convenience samples of commonly consumed foods were purchased from supermarkets in Dallas in 2009–2010. Food samples included a wide variety of lipid-rich foods: fish, peanut butter, poultry, pork, and beef. Thirty-six individual food samples were collected in 2010 and analyzed for α-, β-, and γ-HBCD stereoisomers using liquid chromatography–tandem mass spectrometry (LC-MS/MS). Ten pooled food samples previously collected in 2009 for a study of total HBCD levels using gas chromatography–mass spectrometry (GC-MS), were reanalyzed for α-, β-, and γ-HBCD stereoisomers using LC-MS/MS.

Results: Of the 36 measured individual foods, 15 (42%) had detectable levels of HBCD. Median (ranges) of α- and γ-HBCD concentrations were 0.003 (< 0.005–1.307) and 0.005 (< 0.010–0.143) ng/g wet weight (ww), respectively; β-HBCD was present in three samples with a median (range) of 0.003 (< 0.005–0.019) ng/g ww. Median levels (range) for α-, β-, and γ-HBCD, in pooled samples were 0.077 (0.010–0.310), 0.008 (< 0.002–0.070), and 0.024 (0.012–0.170) ng/g ww, respectively.

Conclusions: α-HBCD was detected most frequently and at highest concentrations, followed by γ-, and then β-HBCD, in food samples from Dallas, Texas. Food may be a substantial contributor to the elevated α-HBCD levels observed in humans. These data suggest that larger and more representative sampling should be conducted.

Hexabromocyclododecane (HBCD) is a brominated aliphatic cyclic hydrocarbon flame retardant (BFR) used in polystyrene foam for thermal insulation in buildings, upholstery textiles, and electrical equipment (Covaci et al. 2006). HBCD is a major BFR produced globally, and before the phase-out of certain polybrominated diphenyl ethers (PBDEs), it was the third most abundant BFR in North America, after tetrabromobisphenol A and PBDEs (Johnson-Restrepo et al. 2008). HBCD enters the environment during its production and by leaching from consumer products (Covaci et al. 2006). Human exposure occurs through dust inhalation and dust and food ingestion (Abdallah and Harrad 2009; Schecter et al. 2009; Thomsen et al. 2008). HBCD is hydrophobic and lipophilic, and it bioaccumulates with half-lives of 2, 60, and 240 days in air, water, and sediment, respectively (Abdallah and Harrad 2009; Germer et al. 2006; Haukås et al. 2009; Johnson-Restrepo et al. 2008; Kunisue et al. 2007; Thomsen et al. 2008; Törnkvist et al. 2011). Terminal elimination half-lives in nonoccupationally exposed humans were calculated to be on average 64 days (range 23–219 days) (Geyer et al. 2004), using the daily intake for total HBCD in adult humans based on a Swedish market basket study (Lind et al. 2002). The authors attributed the high variability observed in half-lives to differences in exposure, total body fat content, and biological differences that affect bioaccumulation, uptake, metabolism, and elimination. Abdallah and Harrad (2011) calculated a human half-life of 165 days for α-HBCD (representing 75% of the maximum half-life of 219 days for the HBCD mixture), whereas a half-life of 55 days was estimated for the β- and γ-isomers (25% of 219 days).

HBCD is categorized as a persistent organic pollutant (POP) because of its persistence, toxicity, and abilities to bioaccumulate and to travel over long distances. It is currently on the European Chemicals Agency candidate list of substances of very high concern (European Chemicals Agency 2010). The U.S. Environmental Protection Agency (EPA) has developed an action plan for HBCD and is considering adding it to its list of “chemicals of concern” (U.S. EPA 2010).

Increased attention has focused on the individual stereoisomers that constitute the HBCD commercial mixture. In laboratory animal studies, individual stereoisomers that compose the commercial mixture have been shown to have different biological properties (Szabo et al. 2010, 2011a). There are three main stereoisomers in the commercial mixture: α-HBCD (10–13%), β-HBCD (1–12%), and γ-HBCD (75–89%) (Heeb et al. 2005). γ-HBCD dominates in the commercial mixture and in the environment, whereas biota show a predominance of α-HBCD (Arsenault et al. 2007; Law et al. 2008). The largest tissue depots found after oral exposure to γ-HBCD in mice included the liver, brain, blood, and fat; however, tissue levels were low because of γ-HBCD’s rapid metabolism and elimination (Szabo et al. 2010). For γ-HBCD, the mouse biological half-life was estimated at 1–4 days with limited capability for bioaccumulation (Szabo et al. 2010). This is in contrast to α-HBCD, which has a biological half-life of 17 days in mice. α-HBCD bioaccumulates and its tissue distribution is dictated by lipophilicity, with higher levels being detected in mouse adipose, liver, muscle, and skin tissues (Szabo et al. 2011a). α-HBCD predominates in biota in part because of the rapid metabolism of γ-HBCD to α-HBCD (Szabo et al. 2010). Toxicity studies suggest that the commercial mixture of HBCD is an endocrine disruptor and developmental neurotoxicant; specifically, HBCD commercial mixtures have been associated with changes in rat thyroid systems (Palace et al. 2010; Saegusa et al. 2009), altered function of human natural killer cells (Hinkson and Whalen 2009, 2010), and neurotoxic effects such as decreased fine manipulative abilities and lower attention in children (Roze et al. 2009). Studies on the health effects of individual stereoisomers are extremely limited. The difficulty of analytical separation, quantities, and purity needed for both *in vivo* and *in vitro* experiments have limited the progress of toxicity studies examining individual HBCD stereoisomers.

Because of the different physical, chemical, and biological properties of the HBCD stereoisomers, there is a growing demand to design studies characterizing individual HBCD stereoisomers. Although studies have investigated HBCD in the environment (Haukås et al. 2009; Law et al. 2008), dust (Harrad et al. 2009), human serum (Roosens et al. 2009), human milk (Ryan et al. 2006), and animal tissues (Johnson-Restrepo et al. 2008; Zegers et al. 2005), few have examined HBCD contamination of food. Such information is notably sparse for foods found in the United States. We previously reported “total HBCD” levels in pooled samples of U.S. food but did not differentiate between stereoisomers (Schecter et al. 2009). Because ingestion is believed to be a major exposure route for HBCD (Covaci et al. 2006), and to examine stereoisomer-specific dietary concentrations from food consumed by the U.S. population, we report here, for the first time, concentrations of α-, β-, and γ-HBCD stereoisomers in selected foods from supermarkets in Dallas, Texas.

## Material and Methods

*Sample collection.* In this study, food samples were collected in Dallas, Texas, in 2009 and 2010. Further study will be needed to determine representative levels for U.S. food in general.

*Analysis of individual food samples collected in 2010 for HBCD stereoisomers used liquid chromatography/electrospray negative ionization with tandem mass spectrometry (LC-MS/MS).* A convenience collection of foods (*n* = 36) was purchased in 2010 from supermarkets in Dallas. The 2010 selection of foods was based on findings of quantifiable HBCD levels in our previous study of total HBCD concentration in U.S. food (Schecter et al. 2009). Food sampled in this analysis included fish, peanut butter, turkey, pork, and beef. After collection, the foods were frozen at –80°C and sent on dry ice to Eurofins Gfa GmbH Laboratory (Hamburg, Germany) for stereoisomer-specific HBCD analysis. Each food sample was individually analyzed for HBCD stereoisomer levels using LC-MS/MS.

*Reanalysis of pooled U.S. food samples collected in 2009 for HBCD stereoisomers used LC-MS/MS.* In our previous study (Schecter et al. 2009), 31 pooled samples from Dallas supermarkets were analyzed for “total HBCD” (Schecter et al. 2009). The foods selected were a convenience sample of commonly consumed foods purchased at local supermarkets, including fish, peanut butter, and poultry. Samples were frozen at –80^o^C and shipped on dry ice to Hamburg, Germany, and stored in a deep freezer in Hamburg until analyzed by gas chromatography–mass spectroscopy (GC-MS). Thirteen of the 31 (42%) pooled samples contained detectable levels of HBCD. Of these 13 pooled samples, 10 had adequate amounts of sample remaining for additional analysis and were chosen for stereoisomer-specific analysis to validate and compare to our previous findings of total HBCD levels. Reanalysis of the 10 pooled 2009 food samples for HBCD stereoisomers was conducted using LC-MS/MS.

*Chemical analyses.* The isotope dilution analysis is a technique used to provide the best precision and accuracy for these chemical analyses. Three native HBCD standards (α-, β-, and γ-HBCD stereoisomers; purity of ≥ 98%) and one carbon-13 (^13^C)-labeled standard (γ-HBCD) were obtained from Wellington Laboratories, (Guelph, Ontario, Canada). The internal standard, ^13^C-labeled γ-HBCD, was added to the homogenized fraction. Soxhlet extraction was performed with hexane:acetone (4:1). The lipid extract was separated, further purified with sulfuric acid, then cleaned up with aluminum oxide (2% water). The final extract was reduced and dried under a light stream of nitrogen and then dissolved in 100 µL methanol for LC-MS/MS analysis. The measurements were performed using LC-MS/MS detection (Varian 1200L LC-MS/MS Triple Quadrupole) with solvents water/acetone/methanol (H_2_O/ACN/MeOH; 20/30/50% vol/vol) [solution A (A)] and ACN/MeOH (30/70% vol/vol) [solution B (B)]. The gradient program consisted of 2 min at 100% (A)/0% (B), ramped to 25% (A)/75% (B) in 13.5 min and held for 4 min, then ramped to 100% (A)/0% (B) in 1 min and held for 6 min. A C18 guard cartridge [4 mm × 2.0 mm inner diameter (i.d.); Phenomenex, Torrance, CA, USA] and a Synergy 4u Fusion RP C-18 column (100 mm × 2.0 mm i.d., 80A; Phenomenex) were used for liquid chromatographic separation.

Quality assurance/quality control measures included a multipoint calibration curve, recalibration of each sequence of analyses (with a minimum of one blank in each batch of a maximum of 10 samples), and duplicate analyses of approximately 20% of the positive samples. Acceptable reproducibility/accuracy was determined by analyzing a laboratory reference sample of pooled fish oil. The ratio between the assigned value and the determined value (i.e., recovery) ranged from 87–110% for α-HBCD (mean 96.5%; relative standard deviation of 8.1%; *n* = 7); and recovery rates ranged between 80–113% for γ-HBCD (mean 93.3%; reproducibility variation coefficient of 10.7%; *n* = 7). The concentration of β-HBCD in this reference sample was below the limit of quantification, and therefore recovery rates were unobtainable.

Total HBCD levels were calculated as the sum of α-, β-, and γ-HBCD stereoisomer levels for each individual food examined, and analyzed with the values below the limit of detection (LOD) set to zero as well as to one-half the LOD (LOD/2). We have elected to report values below the LOD as equal to LOD/2 instead of zero because the results were similar.

*Statistical analysis.* Continuous measures are described using medians, range, and means. To estimate the total distribution of HBCD stereoisomers in U.S. food, we calculated the proportion of the three HBCD stereoisomers in each individually analyzed sample as the mean stereoisomer divided by the mean of the total HBCD with nondetected food values estimated as LOD/2, weighting each food sample equally. We used IBM SPSS software, version 19 (SPSS Inc., Chicago, IL, USA) to analyze these data.

## Results

Table 1 shows stereoisomer-specific HBCD levels for 36 individually analyzed foods purchased in 2010. For all foods except peanut butter, the LODs for each of the HBCD stereoisomers are 0.005 ng/g for α- and β-HBCD and 0.010 ng/g for γ-HBCD (Table 1). LODs for peanut butter were 0.020 ng/g for α- and β-HBCD and 0.040 ng/g for γ-HBCD. Fifteen of 36 individual food samples (42%) had at least one detectable stereoisomer. α-HBCD was present in 13 samples (36%) with a median (range) of 0.003 (< 0.005–1.307) ng/g wet weight (ww); β-HBCD was present in 3 samples (8%) with a median of 0.003 (< 0.005–0.019) ng/g ww; γ-HBCD was present in 8 samples (22%) with a median of 0.005 (< 0.010–0.143) ng/g ww with median values calculated after setting nondetectable levels to LOD/2. Total HBCD levels for these stereoisomers, calculated as the sum of the three stereoisomers, had a median of 0.012 (< LOD to 1.366) ng/g ww. The mean proportion of each HBCD stereoisomer in all 36 samples was 78.0% α-HBCD, 3.8% β-HBCD, and 18.1% γ-HBCD; the mean proportion of each HBCD stereoisomer for the 15 food samples with at least one detectable stereoisomer was 93.6% α-HBCD, 5.3% β-HBCD, and 1.8% γ-HBCD. Of all the foods studied, canned sardines stand out with an α-HBCD level of 1.307 ng/g ww. Smoked turkey sausages had the next highest α-HBCD level of 0.479 ng/g ww. HBCD levels were < LOD for fresh deli meats and fish, chili with beans, and bacon.

**Table 1:** HBCD levels in individual U.S. food samples purchased and analyzed in 2010 using LC-MS/MS (ng/g ww) (*n* = 36).

Stereoisomer levels^a^				
Food	Total^b^	α	β	γ
Sardines in water		1.366		1.307		< 0.005		0.056
Smoked turkey sausages		0.518		0.479		< 0.005		0.036
Fresh salmon, store A		0.446		0.327		< 0.005		0.116
Fresh salmon, store B		0.410		0.346		< 0.005		0.061
Sardines in olive oil #1		0.270		0.262		< 0.005		< 0.010
Fresh catfish, store B		0.162		0.115		0.016		0.031
Fresh deli sliced turkey, store B		0.155		0.135		< 0.005		0.017
Fresh tilapia, store A		0.148		< 0.005		< 0.005		0.143
Chili with beans #1		0.105		< 0.005		< 0.005		0.100
Sardines in olive oil #2		0.067		0.059		< 0.005		< 0.010
Fresh deli sliced ham, store C		0.051		0.027		0.019		< 0.010
Smoked turkey sausages #1		0.049		0.029		0.015		< 0.010
Creamy peanut butter #1		0.040		< 0.020		< 0.020		< 0.040
Creamy peanut butter #2		0.040		< 0.020		< 0.020		< 0.040
Creamy peanut butter #3		0.040		< 0.020		< 0.020		< 0.040
Smoked turkey sausages #2		0.032		0.024		< 0.005		< 0.010
Fresh catfish, store A		0.016		0.008		< 0.005		< 0.010
Fresh catfish, store C		0.014		0.006		< 0.005		< 0.010
Chili with beans #2		0.010		< 0.005		< 0.005		< 0.010
Chili with beans #3		0.010		< 0.005		< 0.005		< 0.010
Bacon #1		0.010		< 0.005		< 0.005		< 0.010
Bacon #2		0.010		< 0.005		< 0.005		< 0.010
Bacon #3		0.010		< 0.005		< 0.005		< 0.010
Fresh deli sliced beef, store C		0.010		< 0.005		< 0.005		< 0.010
Fresh deli sliced turkey, store C		0.010		< 0.005		< 0.005		< 0.010
Fresh deli sliced chicken, store C		0.010		< 0.005		< 0.005		< 0.010
Fresh deli sliced beef, store A		0.010		< 0.005		< 0.005		< 0.010
Fresh deli sliced ham, store A		0.010		< 0.005		< 0.005		< 0.010
Fresh deli sliced turkey, store A		0.010		< 0.005		< 0.005		< 0.010
Fresh deli sliced chicken, store B		0.010		< 0.005		< 0.005		< 0.010
Fresh tilapia, store C		0.010		< 0.005		< 0.005		< 0.010
Fish sticks #1		0.010		< 0.005		< 0.005		< 0.010
Fish sticks #2		0.010		< 0.005		< 0.005		< 0.010
Fish sticks #3		0.010		< 0.005		< 0.005		< 0.010
Fresh tilapia, store C		0.010		< 0.005		< 0.005		< 0.010
Fresh deli sliced beef, store C		0.010		< 0.005		< 0.005		< 0.010
Median		0.012		0.003		0.003		0.005
Mean		0.114		0.089		0.004		0.021
aLODs based on a 20-g sample size. bTotal HBCD levels calculated as sum of stereoisomers with values < LOD set to LOD/2; mean and median values also calculated after setting values < LOD to LOD/2.								

Table 2 presents stereoisomer-specific analysis of 10 pooled food samples purchased in 2009, as well as total HBCD levels previously measured by GC-MS (Schecter et al. 2009). Total HBCD levels as measured by GC-MS and total levels calculated by adding individual stereoisomer levels by LC-MS/MS were similar, with < 0.051-ng/g ww difference between estimates for 9 of the 10 samples. Both α- and γ-HBCD were detected in all 10 analyzed samples, whereas β-HBCD was detected in 8 of 10 analyzed samples, with nondetectable levels of 0.004 and 0.002 ng/g for fresh catfish and canned chili, respectively. Levels for α-, β-, and γ-HBCD [median (range)] were 0.077 (0.010–0.310), 0.008 (< 0.002–0.070), and 0.024 (0.012–0.170) ng/g ww, respectively, with median values calculated after setting nondetectable levels to LOD/2. Total levels as measured by GC-MS were higher than those calculated from LC-MS/MS with two exceptions: *a*) sliced ham, where the totals calculated from LC-MS/MS were higher, and *b*) canned chili, where the totals were equal.

**Table 2:** HBCD levels in U.S. food samples purchased in 2009 (Schecter et al. 2009) analyzed using GC-MS and reanalyzed using LC-MS/MS in 2010 (*n* = 10) (ng/g ww).

Total^a^ (GC-MS)^b^	Total (LC-MS/MS)	Stereoisomer levels				
		Food	α	β	γ
Canned sardines		0.593		0.550		0.310		0.070		0.170
Fresh salmon		0.352		0.123		0.099		0.005		0.019
Peanut butter		0.300		0.282		0.223		0.007		0.052
Fresh tilapia		0.180		0.130		0.090		0.010		0.030
Sausages		0.151		0.119		0.078		0.013		0.028
Fresh catfish		0.133		0.109		0.050		< 0.004c		0.057
Sliced turkey		0.124		0.113		0.076		0.019		0.018
Sliced chicken breast		0.098		0.048		0.025		0.008		0.015
Sliced ham		0.024		0.036		0.012		0.006		0.018
Canned chili		0.023		0.023		0.010		< 0.002c		0.012
Median		0.142		0.116		0.077		0.008		0.024
aTotal HBCD levels measured by laboratory and not calculated as sum of stereoisomers. bData from Schecter et al. 2009. cValues < LOD.										

Table 3 lists available data on stereoisomer-specific HBCD levels (reported on a wet-weight basis) in foods worldwide. Although comparative data for specific foods in each country are not available, it is notable that Romania (0.040–0.250 ng/g ww), Sweden (0.005–0.630 ng/g ww), United Kingdom (0.104–0.730 ng/g ww), and Norway (0.015–1-0.769 ng/g ww) have reported some of the lowest total HBCD levels (Dirtu and Covaci 2010; Driffield et al. 2008; Knutsen et al. 2008; Törnkvist et al. 2011), and the Netherlands has reported the highest seafood total HBCD level (0.200–230.0 ng/g ww) (van Leeuwen and de Boer 2008). Higher HBCD levels have been measured in food samples from the Netherlands, Japan, and Scotland (Fernandes et al. 2008; Nakagawa et al. 2010; van Leeuwen and de Boer 2008) than in samples tested in the present study.

**Table 3:** HBCD levels [range (ng/g ww)] in food worldwide.

HBCD levels				
Country	Food	α	β	γ	Total	Reference
USA		Seafood, meat, peanut butter		0.006–1.307		0.006–0.070		0.012–0.170		0.010–1.366		Present study
Japan		Seafood		0.010–18.30		0.010–2.440		0.020–56.60		0.010–77.30		Nakagawa et al. 2010
Norway		Seafood		0.005–0.653		0.003–0.063		0.007–0.053		0.015–0.769		Knutsen et al. 2008
United Kingdom		Meat, sugars, vegetables, fruits, nuts		0.055–0.290		0.030–0.320		0.019–0.120		0.104–0.730		Driffield et al. 2008
The Netherlands		Seafood		NA		NA		NA		0.200–230.0		van Leeuwen and de Boer 2008
Scotland		Seafood		NA		NA		NA		0.030–12.10		Fernandes et al. 2008
Romania		Meat, dairy, oil		NA		NA		NA		0.040–0.250		Dirtu and Covaci 2010
Sweden		Fish, meat, dairy, eggs, fats		NA		NA		NA		0.005–0.630		Törnkvist et al. 2011
NA, not available.												

## Discussion

Although γ-HBCD is the dominant stereoisomer in commercial HBCD mixtures, α-HBCD was the main stereoisomer found in 23 of 46 analyses (10 composite and 13 individual) as has also been reported in other food studies and in biota (Driffield et al. 2008; Knutsen et al. 2008; Law et al. 2008; Nakagawa et al. 2010). This stereoisomer shift from what is in commercial mixtures has been hypothesized to be due to several reasons, including increased thermodynamic stability (Heeb et al. 2010), biological stability, and persistence of the α-HBCD stereoisomer (Szabo et al. 2011a) as compared to γ-HBCD. Rapid metabolism of γ-HBCD *in vivo* (Szabo et al. 2010) and *in vitro* (Zegers et al. 2005), along with biological stereoisomerization of γ-HBCD to α-HBCD (Szabo et al. 2010), is also suspected to be a contributing factor to the decreased levels of γ-HBCD and increased levels of α-HBCD in biota, and subsequently animal-based foods consumed in the United States and elsewhere.

Stereoisomer-specific concentrations of HBCD measured in this U.S. food study were lower than HBCD levels measured in food samples from some other countries (Fernandes et al. 2008; Nakagawa et al. 2010; van Leeuwen and de Boer 2008). Some of the foods tested for our study may not have been as lipid rich as the foods tested for other studies (for example, certain types of fish), which could account for some disparities (Hayward et al. 2006). Higher HBCD food levels reported in other studies may also be due to a greater use of HBCD in those countries (Morose 2006). In the present study, we did not note an association between higher lipid levels and higher HBCD levels (results not shown).

HBCD stereoisomer levels reported for the present study and for comparisons with other studies are reported on a wet weight basis, which is more representative of food as eaten then food levels reported on a lipid basis (Goscinny et al. 2011). In contrast, we and many authors report measured values of lipid-soluble compounds in biologic samples from humans on a lipid basis to reflect exposure and body burden. In comparing studies it is important to note whether the data is reported on a wet weight or lipid basis because levels of the lipophilic HBCDs will be higher when reported on a lipid basis. Several other studies have also reported evidence suggesting that lipid-rich fish and meats are a major source of HBCD exposure from foods (Dirtu and Covaci 2010; Driffield et al. 2008; Fernandes et al. 2008; Knutsen et al. 2008; Nakagawa et al. 2010; Törnkvist et al. 2011; van Leeuwen and de Boer 2008). However, it is also becoming clear that HBCD contamination is not restricted to fish or meat.

LC-MS/MS measurements confirmed the high levels of HBCD previously reported in a sample of peanut butter assayed in 2009 using GC-MS (300 and 282 ng/g ww, respectively) (Schecter et al. 2009). Driffield et al. (2008) also detected HBCD in nuts bought in the United Kingdom. Individual PBDE and polychlorinated biphenyl (PCB) congeners, pesticides, and a number of perfluorinated compounds (PFCs) were found at much lower levels than HBCD in the same pooled peanut butter samples (Schecter et al. 2009, 2010a). However, HBCD stereoisomer levels were nondetectable in the three individual peanut butter samples tested for the present study. There are many ways that the differing levels found in individual and pooled peanut butter samples may be explained. HBCD found in dust may contaminate foods during preparation in the indoor environment (Abdallah and Harrad 2009). Also, HBCD in soil has been shown to be transferred into vegetables in some instances (Li et al. 2011). Heavy metal accumulation in plants can serve as an indicator of soil contamination; chromium, copper, and zinc have been transferred to and accumulated in the roots and leaves of peanut plants grown in soils contaminated with those metals (Ching et al. 2008). It may be possible that uptake of HBCD from soil by peanuts from select regions might have contributed to HBCD levels measured in this study. The distribution of HBCD in other plant tissues is also found to be stereoisomer specific, with higher levels of α-HBCD in stems and leaves and higher levels of γ-HBCD in roots (Li et al. 2011).

Food contamination levels seen in this study and in our previous study (Schecter et al. 2009) differed among samples of the same type of food, for example among different samples of turkey sausages. This may reflect differences in the source of the foods, differences in handling, and dissimilarities in ingredients. Although some variation was seen between foods, the variation in levels among food samples purchased in Dallas may be similar to variation likely to be observed in foods from other U.S. locations. The variation might be partially due to the different environments in which the animals are raised and the differences in feeds used, with cattle being primarily raised outdoors and swine and poultry primarily coming from indoor environments. Thus, it is fair to say that husbandry practices and feed likely influence animal concentrations of HBCD, but packaging may also contribute to the levels detected.

The data we present here concerning HBCD stereoisomers (α-, β-, and γ-HBCD), and data on perfluorinated compounds (PFCs), PCBs, pesticides, and PBDEs in the same pooled food samples collected in 2009 (Schecter et al. 2009, 2010b), document multiple chemical contamination of U.S. food with various POPs and endocrine disrupting compounds. Interestingly, concentrations of HBCDs are found at higher levels than PCBs, pesticides, and PFCs in those pooled foods measured, and exceeded PBDEs in samples of tilapia, ham, sliced chicken breast, sliced turkey, and peanut butter (Schecter et al. 2009, 2010a). When comparing the total HBCD levels from the 2009 study with total HBCD levels calculated in this study, the total HBCD levels between the two studies are similar, i.e., consistent with the use of different assay methods. It is noteworthy that calculated HBCD intake previously reported for Dallas, Texas, foods of 15.3 ng/day, 2.1 × 10^–7^ mg/kg-bw/day for a 70-kg individual (Schecter et al. 2009), is < 10 mg/kg-bw/day, which is the no observed adverse effect level in rats after exposure to the HBCD commercial mixture. This level is used as the critical effect level by the European Union to characterize risk (European Commission 2008). To our knowledge, human-based benchmarks are not available.

The toxicity of mixtures of both classical and emerging POPs reported in this study and previously is unknown (Schecter et al. 2010a, 2010b). An analysis of a larger and more representative sampling of Texas and other U.S. foods for a wide range of POPs, including HBCD stereoisomers, is indicated. Exposure and health outcome research should continue because of the lack of data regarding levels of HBCD in food, human dietary and other intake levels, and health effects. Special attention to the effects of HBCD stereoisomers from food is warranted for the developing young because total body burdens in infantile mice are 10- and 2.5-times higher than adult levels after exposure to γ-HBCD and α-HBCD, respectively (Szabo et al. 2011b). These differences would lead to higher concentrations of the HBCD stereoisomers in target tissues during critical windows of development. Further research on the toxicity of individual HBCD stereoisomers and mixtures of these, other POPs, and other toxic chemicals detected in food, is strongly indicated.

Finding HBCD in food from our previous Texas food study and HBCD stereoisomers in this study suggests that contamination of U.S. food with HBCD is ongoing (Schecter et al. 2009). These data also suggest that larger and more representative sampling of U.S. food should be conducted. Future studies should quantify to what extent U.S. foods are contaminated with HBCD stereoisomers, estimate what the toxicity of each and all may be, and fully elucidate the mechanisms of stereoisomer shifts for HBCD in biota and the environment.
